# Rapid and cost-effective molecular karyotyping in wheat, barley, and their cross-progeny by chromosome-specific multiplex PCR

**DOI:** 10.1186/s13007-024-01162-x

**Published:** 2024-03-05

**Authors:** Mohammad Ali, Dávid Polgári, Adél Sepsi, Levente Kontra, Ágnes Dalmadi, Zoltán Havelda, László Sági, András Kis

**Affiliations:** 1 Institute of Genetics and Biotechnology, Hungarian University of Agriculture and Life Sciences, Gödöllő, 2100 Hungary; 2 Doctoral School of Plant Sciences, Hungarian University of Agriculture and Life Sciences, Gödöllő, 2100 Hungary; 3 Centre for Agricultural Research, Hungarian Research Network, Martonvásár, 2462 Hungary; 4 Agribiotechnology and Precision Breeding for Food Security National Laboratory, Plant Biotechnology Section, Hungarian University of Agriculture and Life Sciences, Gödöllő, 2100 Hungary; 5 Agribiotechnology and Precision Breeding for Food Security National Laboratory, Plant Biotechnology Section, Centre for Agricultural Research, Martonvásár, 2462 Hungary; 6 Present Address: Institute of Experimental Medicine, Bioinformatics Core Facility, Hungarian Research Network, Budapest, 1083 Hungary

**Keywords:** Cereal, Genotyping, Interspecific hybridization, Molecular marker, Primer design

## Abstract

**Background:**

Interspecific hybridisation is a powerful tool for increasing genetic diversity in plant breeding programmes. Hexaploid wheat (*Triticum aestivum*, 2*n* = 42) × barley (*Hordeum vulgare*, 2*n* = 14) intergeneric hybrids can contribute to the transfer of agronomically useful traits by creating chromosome addition or translocation lines as well as full hybrids. Information on the karyotype of hybrid progenies possessing various combinations of wheat and barley chromosomes is thus essential for the subsequent breeding steps. Since the standard technique of chromosome in situ hybridisation is labour-intensive and requires specific skills. a routine, cost-efficient, and technically less demanding approach is beneficial both for research and breeding.

**Results:**

We developed a Multiplex Polymerase Chain Reaction (MPCR) method to identify individual wheat and barley chromosomes. Chromosome-specific primer pairs were designed based on the whole genome sequences of ‘Chinese Spring’ wheat and ‘Golden Promise’ barley as reference cultivars. A pool of potential primers was generated by applying a 20-nucleotide sliding window with consecutive one-nucleotide shifts on the reference genomes. After filtering for optimal primer properties and defined amplicon sizes to produce an ordered ladder-like pattern, the primer pool was manually curated and sorted into four MPCR primer sets for the wheat A, B, and D sub-genomes, and for the barley genome. The designed MPCR primer sets showed high chromosome specificity *in silico* for the genome sequences of all 18 wheat and barley cultivars tested. The MPCR primers proved experimentally also chromosome-specific for the reference cultivars as well as for 13 additional wheat and four barley genotypes. Analyses of 16 wheat × barley F1 hybrid plants demonstrated that the MPCR primer sets enable the fast and one-step detection of all wheat and barley chromosomes. Finally, the established genotyping system was fully corroborated with the standard genomic in situ hybridisation (GISH) technique.

**Conclusions:**

Wheat and barley chromosome-specific MPCR offers a fast, labour-friendly, and versatile alternative to molecular cytogenetic detection of individual chromosomes. This method is also suitable for the high-throughput analysis of distinct (sub)genomes, and, in contrast to GISH, can be performed with any tissue type. The designed primer sets proved to be highly chromosome-specific over a wide range of wheat and barley genotypes as well as in wheat × barley hybrids. The described primer design strategy can be extended to many species with precise genome sequence information.

**Supplementary Information:**

The online version contains supplementary material available at 10.1186/s13007-024-01162-x.

## Background

Consistent human selection has accumulated useful agronomic traits in the allohexaploid wheat (2*n* = 6*x* = 42, AABBDD) genome. However, due to the prevention of homoeologous pairing (and recombination) between its sub-genomes [1, 2], the allelic diversity of these traits cannot be fully unlocked and exploited for the genetic improvement of wheat. Of the several alternatives to increase genetic diversity in wheat, interspecific hybridisation can be an efficient tool to further extend the existing range of useful traits such as biotic or abiotic stress tolerance [3]. As an example, the present cultivars of triticale (× *Triticosecale* sp. Wittmack ex A. Camus) combine the high yield of wheat with the adaptation of rye (*Secale cereale* L.) to abiotic stress allowing this man-made cereal to be grown successfully on a larger surface than ever before [4]. The hybridisation of wheat with barley (2*n* = 2*x* = 14, HH genome), too, promises the transfer of agronomically useful genes to wheat by creating chromosome addition and translocation lines or even full hybrids [5]. As a result of early uniparental genome elimination and its genotype dependence, 20–90% of the F1 generation can be maternal haploid wheat. The rest of the F1 will contain wheat and barley chromosomes in various combinations [6]. Due to the random chromosome composition, it is essential to determine the individual karyotype of the F1 plants and their progeny. To this end, a standard cytological technique is Genomic In Situ Hybridisation (GISH: [7, 8]), which allows the detection of translocations as well as chromosome additions and substitutions [9]. In situ hybridisation also applies labelled DNA sequences as probes that provide specific hybridisation patterns on individual chromosomes, allowing their precise identification. While detailed cytogenetic information can be obtained by in situ hybridisation techniques, they are time-consuming and require special tissues (young roots or anthers), which hampers the routine and large-scale testing of breeding lines [10]. Due to the relatively high number of chromosomes (between *n* = 3-4*x* = 21–28) present in wheat × barley F1 hybrids, their individual detection could be complicated, so there is a need for an efficient, easy-to-implement, and easy-to-evaluate screening technique. Chromosome-specific DNA markers offer a good alternative because sampling can be carried out from any tissue type and at any developmental stage.

Multiple attempts were made earlier to detect barley chromosomes in a wheat background using RFLP [11, 12], AFLP [13], and EST [14, 15] markers but mainly for single chromosomes [16–20], not systematically for all wheat and barley chromosomes in an easy multiplex format.

In the present work, we have developed a Multiplex PCR (MPCR) method to identify each of the seven homoeologous chromosomes from each (sub-)genome (A, B, D, and H) in wheat, barley, and their hybrids. Via a bioinformatic analysis of wheat and barley reference genomes, we created a large set of unique primer pairs for individual chromosomes and verified their specificity to a wide range of wheat and barley cultivars. The selected primer sets also proved to be highly specific simultaneously for all the chromosomes of the individual sub-genomes in wheat × barley hybrids. This chromosome specificity was partially preserved even among more distantly related species belonging to the genera *Triticum* and *Hordeum*. Our workflow of primer design is suitable for adaptation to other agriculturally important species in which interspecific crossing is applied for their genetic improvement.

## Materials and methods

### Plant material and DNA extraction

F1 hybrids were produced by crossing M1, a doubled maternal haploid of ‘Sichuan’ [21] hexaploid spring wheat (*Triticum aestivum*, 2*n* = 6*x* = 42, AABBDD) as a female with the two-row spring barley ‘Golden Promise’ (*Hordeum vulgare*, 2*n* = 2*x* = 14, HH) as a male partner. Parental plants were kept in a reach-in growth chamber (Conviron, Winnipeg, Canada) under 13–15 h photoperiod (150–500 µmol m^− 2^ s^− 1^ PPFD) and constant 18 °C temperature, while the synchronisation of flowering periods was achieved with successive plantings. Crossings and subsequent embryo rescue at 14 days after pollination were performed as described [21]. Plantlets of all other wheat and barley genotypes (Supplementary Table 1: right panel) used for MPCR analyses were grown in peat blocks.

Total DNA was obtained from young leaf and root samples with a direct DNA extraction method. Leaf pieces of approximately 5 × 5 mm or root pieces of approximately 2 cm were placed into a 1.5 mL Eppendorf tube containing 100 µL of Extraction solution (E7526-24ML, Sigma-Aldrich, St. Louis, MO, USA) together with a stainless-steel bead (3 mm diameter, Qiagen Sciences, Germantown, MD, USA). The samples were homogenised in a mixer mill (Bullet Blender Storm Pro, Next Advance, Troy, NY, USA) at speed grade 8 for 30 s. The mixture was incubated at 95 ℃ for 15 min in a dry heat block followed by cooling on ice for 1 min. Finally, 100 µL of Dilution solution (D5688-12ML, Sigma-Aldrich) was added, and after vortexing, the samples were spun for 1 min at 18,000 × g. The supernatant (100 µL) was transferred into a new 1.5 mL Eppendorf tube and the DNA was stored at -20 ℃ until required.

### Generation of multiplex PCR primers for the wheat and barley genomes (Fig. 1)

**Fig. 1:**
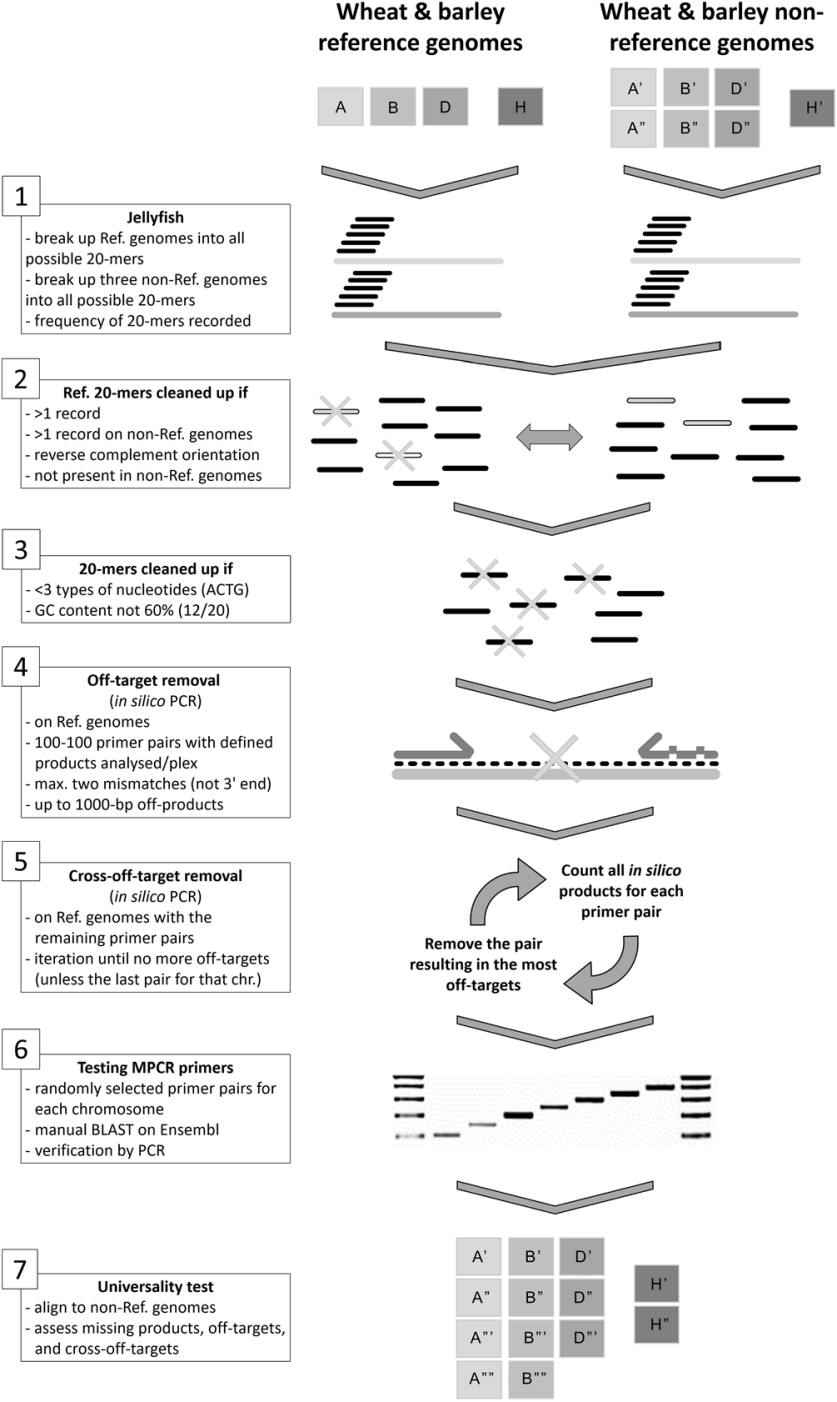
Schematic presentation of the refined protocol for the design of chromosome-specific MPCR primers

First, the reference genomes of wheat (*T. aestivum* ‘Chinese Spring’, IWGSC RefSeq v1.0) and barley (*H. vulgare* ‘Golden Promise’, GPv1) were downloaded from Ensembl (Supplementary Table 1: left panel). Both reference genomes were broken down to 20-mers (all possible 20-bp long sequences) with Jellyfish [22]. These 20-mers were then compared with 20-mers obtained from three additional genome assemblies of ‘Weebill 1’ and ‘Claire’ wheat cultivars and the reference genome (V3) of ‘Morex’ barley (Supplementary Table 1: left panel). Twenty-mers that occurred more than once in either of the genomes or appeared in all genomes as exact matches in either orientation were discarded. Then, 20-mers containing less than three types of nucleotides were also removed and the ones with 60% GC content were selected (Fig. 1: steps 1–3).

Since primers can anneal incompletely during PCR, 100 pairs per chromosome were randomly selected and mapped to the reference genomes with PatMaN [23] by allowing two mismatches. Putative primer pairs that had matched the target genome more than once within 1000 bp in both orientations were removed. The remaining primer pairs with a specific distance between them were selected for each chromosome so that the amplified fragments could be grouped as follows (± 5 bp): chr1–100 bp, chr2–150 bp, chr3–200 bp, chr4–250 bp, chr5–300 bp, chr6–350 bp, and chr7–400 bp. These primer pairs were then collected into four pools: plex-A, plex-B, and plex-D for the wheat A, B, and D sub-genomes, respectively, and plex-H for the barley genome. To analyse the cross-off-target effect, all primer pairs for each plex group were evaluated *in silico*, and the pairs with the highest number of unspecific products were iteratively removed until no cross-off-targets remained (Fig. 1: steps 4–5).

We randomly selected a set of primer pairs for each chromosome and verified them manually by Ensembl BLAST. To improve specificity, the primer positions were occasionally shifted by a couple of nucleotides or their lengths were increased in a few cases. After individual PCR experiments of the selected primer pairs on the reference genomes (Fig. 1: step 6), the more universal applicability was checked *in silico* for each plex by including an additional 16 bread wheat [24] and two barley genome assemblies (CAJHDD01, PRJEB34496) as well as those of progenitor and wild species (Fig. 1: step 7, Supplementary Table 1: left panel).

### PCR and multiplex PCR assay

The single PCRs were performed in 20 µL volumes containing 1 µL of direct total DNA extract, 4 µL of 5X Phusion Green HF Buffer (F-538, Thermo Scientific, Waltham, MA, USA), 0.5 µM of each forward and reverse primer, 4 µM dNTPs (Thermo Scientific), 0.4 U Phusion Hot Start II High-Fidelity DNA Polymerase (F-549, Thermo Scientific) and water to the final volume. The PCR cycling conditions were as follows: 98 ℃, 3 min; 32x [98 ℃, 10 s; 65 ℃, 15 s; 72 ℃, 10 s]; 72 ℃, 10 min; 4 ℃ hold.

The Multiplex PCR (MPCR) assay was performed for the seven chromosomes of each wheat sub-genome (A, B, and D) and of the barley H genome using the 2X Phusion U Green Multiplex PCR Master Mix (F-564, Thermo Scientific). MPCRs were carried out in a total volume of 20 µL consisting of 10 µL of master mix, 0.3 µM of each primer, 1 µL of total DNA extract, and water to the final volume. In some experiments (Fig. 3B), we used the 5X Phusion Green HF Buffer with Phusion Hot Start II High-Fidelity DNA Polymerase, or Phire Hot Start II DNA Polymerase (F-122, Thermo Scientific) either with its 5X Phire Green Reaction Buffer (F-527, Thermo Scientific) or the 5X Phusion Green HF Buffer as in the single PCRs except for the primer concentrations, which were at 0.3 µM for each. MPCR cycling conditions for the Phusion U Green Multiplex PCR Master Mix were as follows: 98 ℃, 3 min; 32x [98 ℃, 10 s; 65 ℃, 30 s; 72 ℃, 10 s]; 72 ℃, 10 min; 4 ℃ hold for plex-A, plex-B, and plex-D. For plex-H, the annealing temperature was modified to 68 ℃. For all other enzyme and buffer combinations, 68 ℃ annealing temperature was constant for all primer plexes.

The PCRs were carried out on a Mastercycler® nexus gradient thermal cycler (Eppendorf, Hamburg, Germany). The amplification products were separated by electrophoresis on 2% (w/v) ethidium bromide-containing agarose gels in 1X TBE (89 mM Tris-borate, 2 mM EDTA, pH 8.3) buffer for 30 min at 130 V. The GeneRuler™ 100 bp Plus DNA Ladder (Thermo Scientific) was the molecular size marker in all gels. Gel images were captured using the ChemiDoc™ MP Imaging System (Bio-Rad Laboratories, Hercules, CA, USA).

### Molecular cytology techniques

Roots of the wheat × barley F_1_ hybrids were harvested from pots for mitotic chromosome preparations. The collected roots were pre-treated in ice-cold water (containing melting ice) for at least 24 h. Roots were then fixed in Clarke’s fixative (3:1 v/v mixture of absolute ethanol and glacial acetic acid) for 5 d at 37 °C and stained with 1% (w/v) acetocarmine (C1022, Sigma-Aldrich). After 2 weeks of storage in Clarke’s fluid at -20 °C the root tissue was used to make chromosome preparations.

To produce the GISH probe, the total DNA of barley (‘Morex’) extracted from fresh young leaves using the CTAB method was fragmented for 6 min in a pressure cooker to obtain 300–500 bp long fragments. One µg of fragmented barley DNA was labelled by nick-translation (AF594 NT Labelling Kit, PP-305 L-AF594, Jena Bioscience, Jena, Germany) and applied as a probe at 40–50 ng per slide.

The FISH probe was prepared by PCR amplification of the barley 5 S rDNA coding and flanking noncoding regions [25] followed by direct labelling via nick-translation (AF488 NT Labelling Kit, PP-305 L-AF488, Jena Bioscience).

The detection and identification of barley chromosomes by the GISH and FISH techniques were performed simultaneously as described [26]. Briefly, the chromosome preparations were digested with a 50 mg/mL pepsin-1 mM HCl solution for 1–2 min followed by post-fixation in 4% (w/v) PFA (diluted from 16% stock, 28,908, Thermo Scientific) for 10 min. The final hybridisation mixture consisted of 60% (v/v) deionised formamide (F9037, Sigma-Aldrich), 10% (w/v) dextran sulphate (D8906, Sigma-Aldrich), and 2X Saline Sodium Citrate buffer. Seventeen µL of this hybridisation mixture was applied to each microscope slide containing 40–50 ng of each of the labelled probes and an excess (30:1 to the GISH probe) of unlabelled wheat total DNA to block unspecific signals. The probe mixture was first denatured at 85 °C for 8.5 min, then, after adding to the slides, additionally denatured at 75 °C for 3 min. After the post-hybridisation washings, the slides were covered with 24 × 32 coverslips and 12 µL of Vectashield antifade solution with DAPI (H1200, Vector Laboratories, Burlingame, CA, USA). Images were taken by an SP8 confocal laser scanning microscope (Leica Microsystems GmbH, Wetzlar, Germany) equipped with an HC PL APO CS2 63×/1.40 oil immersion objective.

## Results

### Bioinformatic analysis and design of chromosome-specific MPCR primers

To generate Multiplex PCR (MPCR) primer sets specifically detecting the individual chromosomes in wheat and barley, first we broke two reference genomes into approximately 18.68 billion 20-bp long sequences. We screened for basic parameters, i.e., uniqueness (in exact matches), complexity, and GC content, to find the best primer candidates, and narrowed them down to 35.28 million suitable sequences. To facilitate easy and chromosome-specific detection, sequences with specific distances between them were selected. The obtained 270,413 pairs were sorted into four sub-groups composing the MPCR sets (plex-A, wheat A sub-genome; plex-B, wheat B sub-genome; plex-D, wheat D sub-genome; plex-H, barley genome). We tested several primer pairs by PCR, but we encountered an abundance of unspecific fragments in some cases.

To eliminate this shortcoming, we refined the protocol by using more genomes, allowing mismatches as well as controlling off-targets and cross-off-targets (Fig. 1). First, we discarded 20-mers with no or multiple occurrences in three additional genome assemblies (two wheat and one barley), then they were filtered for complexity, and GC content which reduced the number of potential 20-mer sequences with maximum two mismatches from the original 18.68 billion to 287.29 million. Then, we eliminated primer pairs producing off-target products, selected from the rest the defined size groups, and randomly selected 100 primer pairs for each chromosome from the final 9437 pairs. By modelling the use of all 700 pairs (per plex) in a PCR in the reference genomes allowing two mismatches, we iteratively removed the pairs with the highest number of off-targets until a cross-off-target free final set was reached (Supplementary Table 2). Some chromosomes had very few pairs to choose from, e.g., chromosome 2D had only three pairs, while other chromosomes had many more primer pairs.

**Fig. 2:**
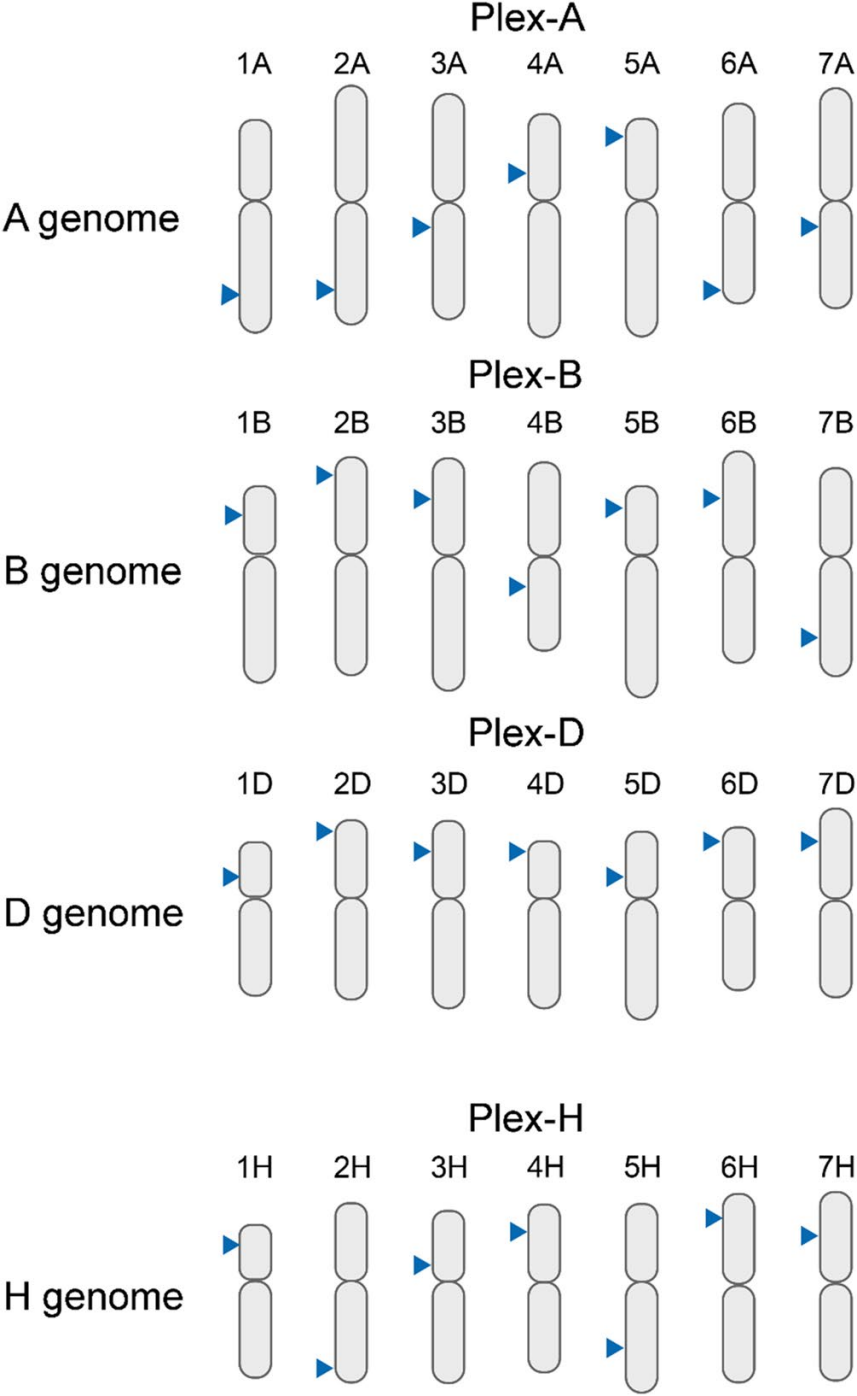
Schematic representation of target chromosomes with the target positions of Multiplex PCR primer sets. The upper three panels represent the positions of specific MPCR primers in seven chromosomes of the wheat A, B, and D sub-genomes (plex-A, plex-B, and plex-D, respectively). The bottom panel shows the positions of specific MPCR primers in the seven barley chromosomes (plex-H). The corresponding chromosome locations are indicated with arrowheads

The selected primer sets (Supplementary Table 3, Fig. 2) were fully confirmed manually by PCR and by cross-referencing *in silico* to the sequenced genomes of 16 bread wheat and two barley cultivars with a few exceptions (Supplementary Table 4). The analysis by wheat-specific 350bp_F_6A and 350bp_R_6A primers revealed the production of two 346 bp amplicons deriving from different locations of the ‘Robigus’ wheat genome. Due to the incomplete genome assembly of this cultivar, this prediction might be a bioinformatic artifact. In the case of 150bp_F_2B and 150bp_R_2B primers, the bioinformatic analysis did not predict the amplicon in the genome of ‘LRPB Lancer’ wheat. However, PCR verification with these primers revealed the accumulation of the expected product using the DNA of this cultivar.

Finally, the primer pair 250bp_F_4D and 250bp_R_4D could result in a 242-bp by-product from chromosome 4B on DNA templates of the wheat cultivars ‘LRPB Lancer’, ‘Paragon’, ‘SY Mattis’, and ‘Julius’. The search for unspecific amplicons revealed a 3’ end mismatch in the 250bp_F_4D primer that prevented the amplification as confirmed by sequence analyses of this PCR product (data not shown).

### Verification of the designed MPCR primer sets

**Fig. 3:**
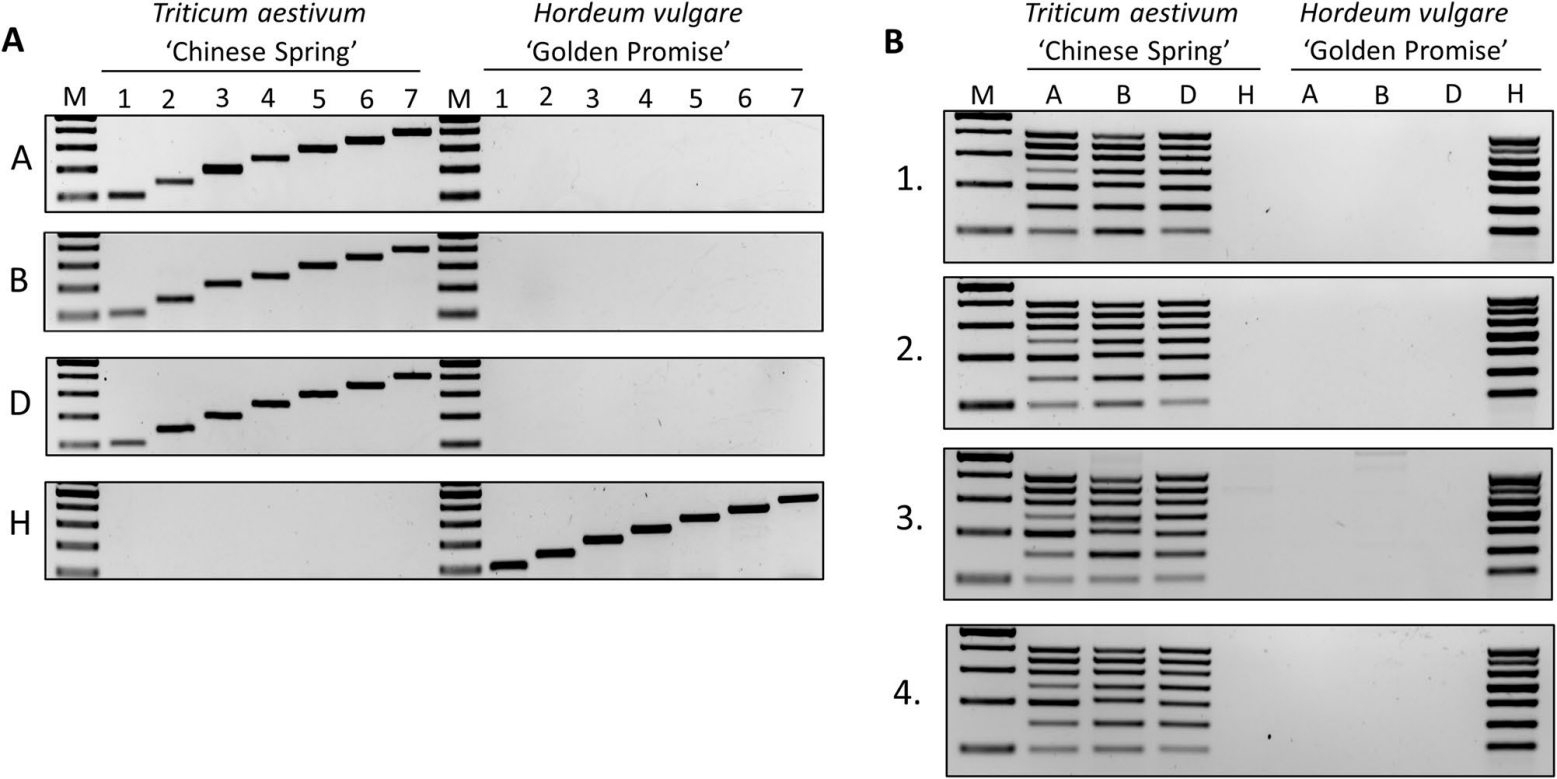
**A** Single PCR amplifications with primers specific to chromosomes 1–7 of the A, B, and D sub-genomes of wheat (‘Chinese Spring’) and the H genome of barley (‘Golden Promise’). 1–7: the PCR amplicons corresponding to each chromosome, M – Molecular size marker (GeneRuler™ 100 bp Plus DNA Ladder). Reaction components: Phusion Green HF buffer and Phusion Hot Start II High-Fidelity DNA Polymerase. **B** MPCR amplification of chromosomes 1–7 of the A, B, and D sub-genomes of wheat (‘Chinese Spring’) and the H genome of barley (‘Golden Promise’) using different buffers and DNA polymerases. 1: Phusion U Green Multiplex PCR Master Mix, 2: Phusion Green HF Buffer using Phusion Hot Start II High-Fidelity DNA Polymerase, 3: Phire Green HF buffer using Phire Hot Start II High-Fidelity DNA Polymerase, 4: Phusion Green HF buffer using Phire Hot Start II High-Fidelity DNA Polymerase. M – Molecular size marker (GeneRuler™ 100 bp Plus DNA Ladder)

The amplicons of various MPCR sets were designed to exhibit product sizes that increased stepwise, characteristic for each chromosome in the (sub-)genomes: chr1 − 100 bp, chr2–150 bp, chr3–200 bp, chr4–250 bp, chr5–300 bp, chr6–350 bp, and chr7–400 bp. To verify the specificity and sensitivity of the primer pairs, first we tested them in individual PCRs on the reference genomes, and products with the expected sizes were obtained in all cases (Fig. 3A). The wheat-specific (‘Chinese Spring’) primers precisely detected the individual wheat chromosomes, and the barley-specific primers showed no cross-reactions with wheat. The chromosome-specificity of PCR products was confirmed by sequence analyses, which revealed that all 28 products corresponded to the sequences predicted with the bioinformatic analyses (Supplementary Table 5). These results prove that the various primer sets are specific for the individual target chromosomes in each genome.

Next, we tested the competency of the chromosome-specific primer sets with the total DNA of the two reference cultivars (‘Chinese Spring’ and ‘Golden Promise’) and arranged the MPCR results according to the (sub-)genomes, which resulted in distinct and clear bands for all chromosomes of wheat and barley (Fig. 3B). The size of the products corresponded to the proper sizes of the single PCRs (compare Fig. 3A and B). No unspecific products and no cross-reaction between wheat and barley DNAs were observed in the MPCRs. The technical flexibility of the elaborated MPCR method was also tested with various buffer-DNA polymerase combinations. All four commercial buffer-polymerase systems equally supported DNA amplification without unspecific products (Fig. 3B). Altogether our data show that the designed primer sets are suitable for performing complex MPCRs to obtain multiple specific products and the execution of MPCRs is technically flexible and economic.

### Wide application of MPCR in a wheat and barley panel

**Fig. 4:**
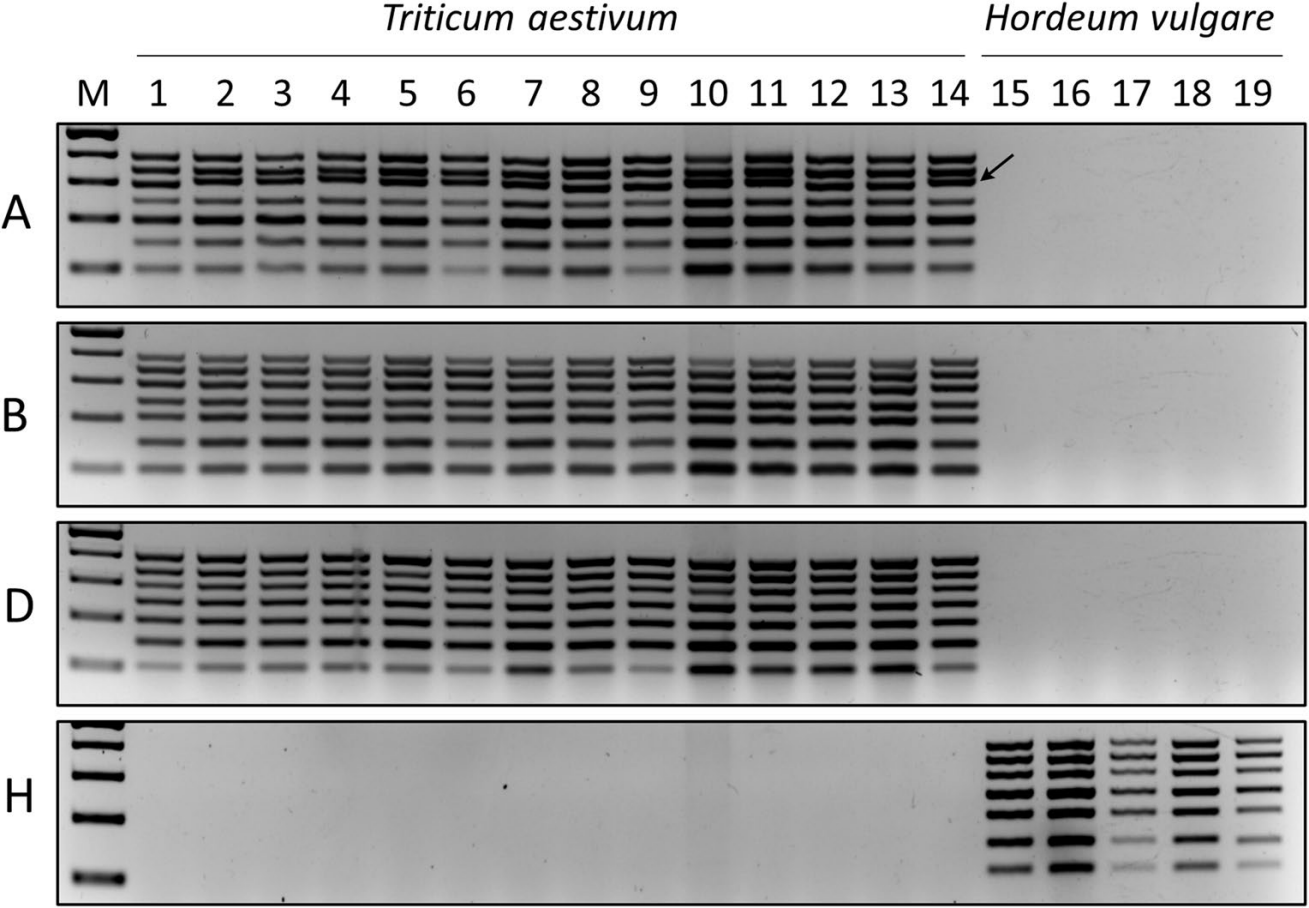
MPCR amplification of chromosomes 1–7 of the A, B, and D sub-genomes of different wheat cultivars (1: Chinese Spring reference genome, 2: Bobwhite, 3: Fielder, 4: Bánkúti 1201, 5: LRPB Lancer, 6: CDC Stanley, 7: Paragon, 8: SY Mattis, 9: Julius, 10: Cadenza, 11: Weebill 1, 12: Claire, 13: Robigus, 14: Jagger) and the H genome of five barley cultivars (15: Golden Promise reference genome, 16: Morex, 17: Igri, 18: California Mariout, 19: Esperanza). Arrow – increased 5 A-specific product sizes. M – Molecular size marker (GeneRuler™ 100 bp Plus DNA Ladder). Reaction components: Phusion Green HF Buffer and Phusion Hot Start II High-Fidelity DNA Polymerase

Total DNA samples extracted from 14 wheat and five barley cultivars (Supplementary Table Supplementary Table 1: right panel) were studied in separate MPCRs for the wheat A, B, and D sub-genomes and the barley H genome (Fig. 4: panels A to H, respectively). The MPCRs resulted in distinct and clearly visible band patterns for all (sub-)genomes at the expected sizes. No unspecific cross-reactions were detected among the tested cultivars. However, the 5 A chromosome-specific amplicon exhibited a minor size increase in the ‘Bobwhite’, ‘Fielder’, ‘Bánkúti 1201’, ‘LRPB Lancer’, ‘CDC Stanley’, ‘Paragon’, ‘Cadenza’, ‘Weebill 1’, and ‘Jagger’ cultivars (Fig. 4: panel A, arrow). *In silico* sequence analyses of these amplicons revealed that they harbour a 12-bp insertion in the corresponding genomic region (Supplementary Table 4). Our data show that the designed MPCR primer sets are highly specific and exhibit no cross-reactions between the genomes of different cultivars and species.

### MPCR primer sets accurately identify the chromosome composition of wheat × barley hybrids

**Fig. 5:**
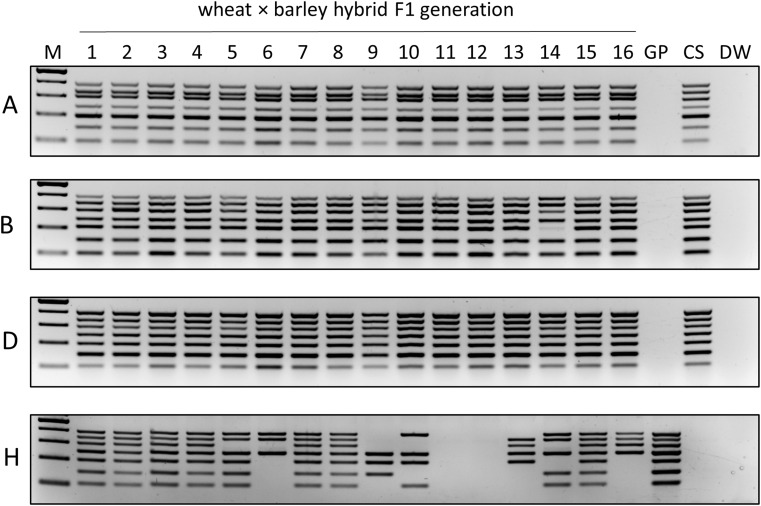
MPCR amplification of chromosomes 1–7 of the A, B, D, and H sub-genomes of wheat × barley hybrids (1–16). M – Molecular size marker (GeneRuler™ 100 bp Plus DNA Ladder), GP – ‘Golden Promise’, CS – ‘Chinese Spring’, DW – no template control. Reaction components: Phusion Green HF Buffer and Phusion Hot Start II High-Fidelity DNA Polymerase

As a practical application, we investigated the designed MPCR primer sets in ‘Sichuan’ wheat (♀) × ‘Golden Promise’ barley (♂) F1 hybrid plants. Sixteen plants were regenerated from 18 embryos rescued from 20 pollinated spikes. MPCR analyses of the obtained plants revealed that they contained the full set of wheat chromosomes and only plant No. 14 exhibited a faint band for the 3B wheat chromosome-specific product (Fig. 5). When testing for the barley chromosomes, seven out of 16 hybrid plants (Nos. 1–4, 7–8, and 15) showed the presence of all barley chromosomes in the MPCRs. For the rest, three (in plant Nos. 6 and 9), four (Nos. 10, 13, and 16), five (No. 14), and six (No. 5) barley chromosomes were maintained and not lost during early development (Fig. 5). Finally, two plants (Nos. 11–12) turned out to be maternal wheat haploids because all barley chromosomes were missing. These results showed that the MPCR primer sets can easily and cost-effectively distinguish the various chromosomes of wheat and barley even in a hybrid background.

**Fig. 6:**
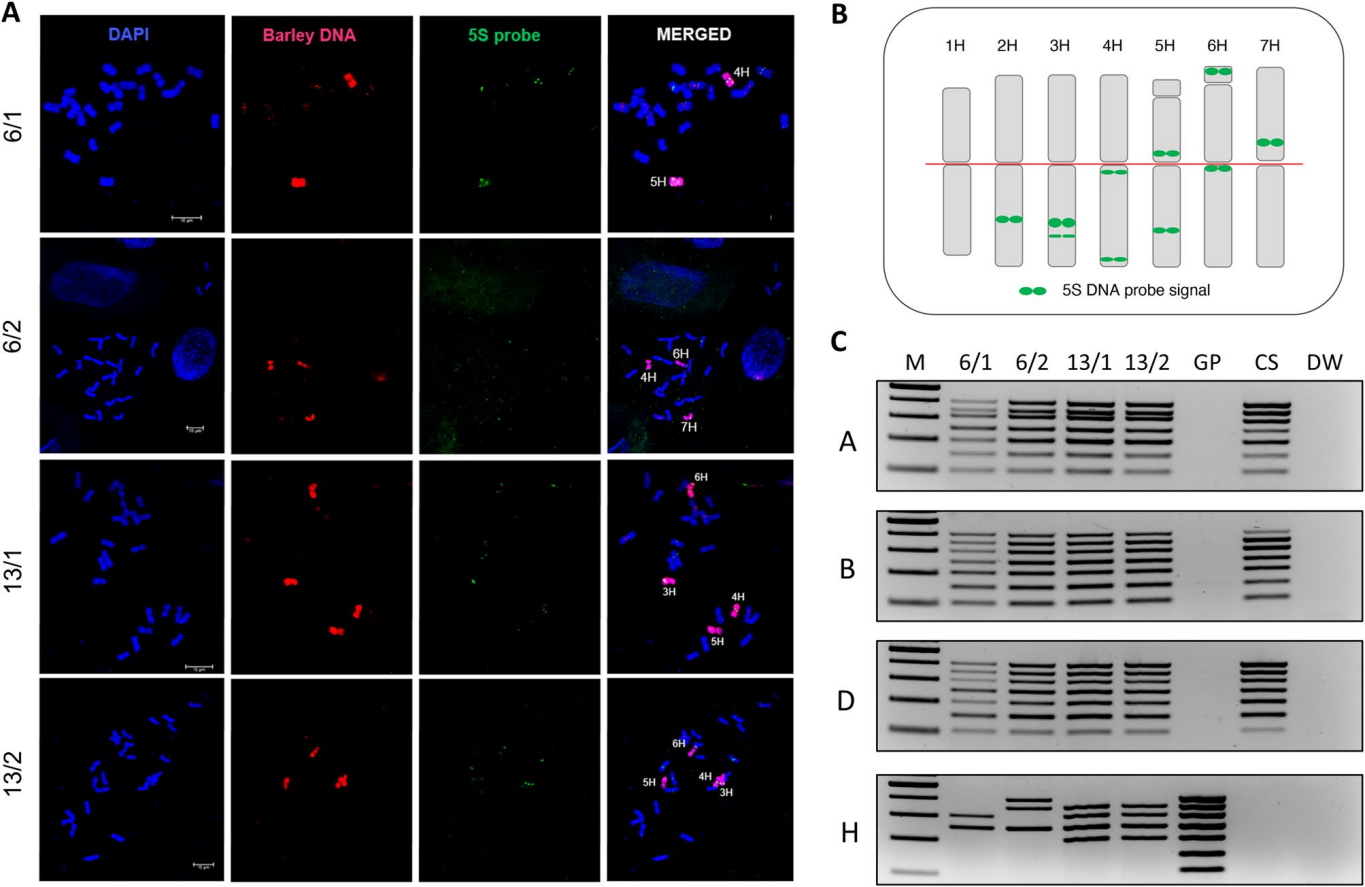
**A** Chromosome in situ hybridisation on two root segments from each of two wheat × barley hybrids (plant No. 6 and 13 on Fig. 4). The barley genome is detected by GISH (red label) and the barley chromosomes are identified by FISH (5 S rDNA, green label). The chromosomes are counterstained with DAPI (blue). Bars = 10 μm. **B** Schematic position of the 5 S rDNA-specific probe on the barley genome (red line, position of the centromere). **C** MPCR amplification of chromosomes 1–7 of the A, B, D, and H sub-genomes of wheat × barley hybrids (same plant Nos. 6 and 13) on DNA templates extracted from the root segments used for GISH (Fig. 5A). M – Molecular size marker (GeneRuler™ 100 bp plus DNA Ladder), GP – ‘Golden Promise’, CS – ‘Chinese Spring’, DW – no-template control. Reaction components: Phusion Green HF Buffer and Phusion Hot Start II High-Fidelity DNA Polymerase

To verify our MPCR technology we selected two hybrid plants (Fig. 5: Nos. 6 and 13) for GISH analysis and FISH using a barley 5 S rDNA-specific probe for the identification of individual barley chromosomes (Fig. 6A and B). Since mitotic chromosomes are usually obtained from root tips, we selected two roots from each plant and simultaneously processed them both for in situ hybridisation and MPCR. According to GISH-FISH analysis, the two root tips of the hybrid plant No. 6 carried the barley chromosomes 4 H + 5 H and 4 H + 6 H + 7 H, respectively (Fig. 6A) as also revealed by MPCR (Fig. 6C), indicating the genetically mosaic nature of this plant. Both root tips of hybrid plant No. 13 contained barley chromosomes 3-6 H, in agreement with the results of MPCR (Fig. 6A and C). Thus, GISH-FISH and MPCR resulted in an identical distribution of barley chromosomes in all four root samples demonstrating that MPCRs are a valuable tool for the fast screening of hybrid plants.

### MPCR analysis of related *Triticum* and *Hordeum species*

To get an impression of the taxonomic limits of the primers’ broader utilisation, we tested them with wild relatives and progenitor species of wheat and barley. The *in silico* alignment of the final primer set with the available seven sequenced genomes (Supplementary Table 1: left panel) revealed perfect homologies and no unspecific products in the genome sequences of *T. spelta* (AABBDD sub-genomes), *T. turgidum* ssp. *durum* (AABB), *T. dicoccoides* (AABB), and *Aegilops tauschii* (DD) as well as of *H. spontaneum* (Supplementary Table 6). On the other hand, only four and two out of the seven chromosome-specific target sites were present in the genomes of *T. urartu* (AA) and *H. marinum* (XaXa), respectively (Supplementary Table 6: red labels).

**Fig. 7:**
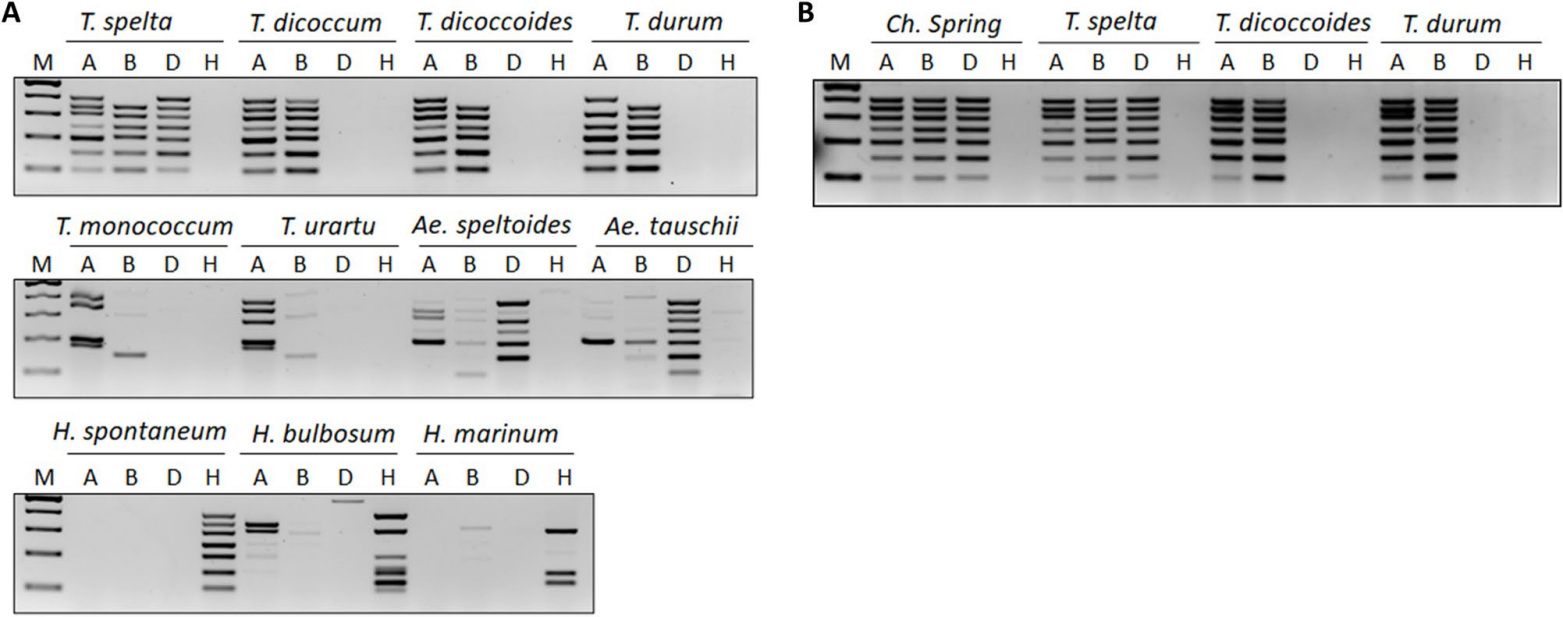
**A** MPCR amplification of chromosomes 1–7 of the A, B, and D sub-genome of wheat and the H genome of barley using various wheat and barley species. **B** Improved MPCR amplification with substituted primer pairs specific for the 6 A and 7B chromosomes. M – Molecular size marker (GeneRuler™ 100 bp Plus DNA Ladder). Reaction components: Phusion Green HF Buffer and Phusion Hot Start II High-Fidelity DNA Polymerase

In contrast to the results obtained with the cultivars, the *in silico* predictions were not fully in line with the MPCR results in the case of the *Triticum* and *Hordeum* species (Fig. 7). In the hexaploid *T. spelta* the 7B chromosome-specific primer pair did not yield any product, similarly to the AABB tetraploid *T. dicoccoides*, although its amplification using *T. dicoccum* (also AABB) was successful. In addition to this 7B chromosome-specific product, in *T. turgidum* ssp. *durum* (AAABB) the 6 A chromosome-specific product was missing, too (Fig. 7A: top panel). The replacement of these two primer pairs with new ones (Supplementary Table 3) resulted in the amplification of correct products (Fig. 7B). The designed chromosome-specific primer pools thus represent useful alternatives and can serve as an additional resource.

The MPCRs performed with the A-genome containing *T. monococcum* and *T. urartu* gave a partial plex-A-specific pattern (four and five products, respectively, Fig. 7A: middle panel) and some unspecific products with the plex-B primers.

Remarkably, in *Ae. speltoides*, the hypothetical donor of the B sub-genome, up to six correct bands were obtained with the plex-D primers besides six faint bands with the plex-B primers as well as some correct-sized products with plex-A primers, too. Conversely, the definite D-genome donor *Ae. tauschii* also produced some bands with plex-A and plex-B primers (Fig. 7A: middle panel).

All plex-H primers worked as expected on *H. spontaneum* (HH genome) the closest relative of cultivated barley. However, the same primers gave only partial results with *H. bulbosum* (HbHb) and the even more distant *H. marianum* (Fig. 7A: bottom panel).

## Discussion

The objective of this work was to develop a Multiplex PCR (MPCR) assay for the identification of individual wheat and barley chromosomes and their reliable tracking in heterogeneous genetic backgrounds and in wheat × barley hybrids. This requirement assumes sufficient sequence divergence between the barley and wheat (sub-)genomes to ensure the finding of genome- and chromosome-specific targets without off-targets between the two species. The general sequence homology between hexaploid wheat and barley as evaluated by reassociation kinetic studies between cross-hybridised genomic DNA [27–30] and by sequencing more than one thousand random ESTs [31] was in the range of 45–60% and ca. 45%, respectively. Since these two lines of data fall in the same range and appear to confirm each other, it can be safely estimated that the overall wheat-barley genome homology may be around 50%. This degree of sequence difference provided ample flexibility for the design and selection of species-specific primer sets.

The issue of interspecific genome homology also emerged when the MPCR assay was extended to the genome progenitor and tetraploid species of wheat. The less precise chromosome identification can be interpreted in light of recent data on the evolutionary history of hexaploid wheat. The uncertain assignment of the markers, especially in the B and D genome progenitors, is consistent with the proposed polyphyletic origin of the donor species [32]. Thorough sequence-based phylogenetic analyses indicated that about 3–4 million years ago, i.e., 1–2 million years before the presumed appearance of the D-genome donor *Ae. tauschii* (2–3 million years ago [33]), hybridisations between the ancestors of the A- and B-genome lineage contributed to the development of the D-genome lineage [34, 35], from which the actual wheat D-genome donor(s) would have emerged later. This scenario should have led to A-B-D inter-genome sequence similarities even among the current wild species as observed in our analysis. Additionally, the ambiguous MPCR results with the wild species may have also been caused by the fact that the single accessions tested were different from the sequenced ones whereas the genetic variation in these ancient species is extremely large.

In contrast, the cultivars’ gene pool carries less genetic variation and the MPCR agreed with the *in silico* prediction due to the many whole genome sequences available. This indicates that the reliability of the MPCR approach will be significantly improved and extended taxonomically when a higher number of accessions in the *Triticum* and *Hordeum* species are fully sequenced.

The GISH technique has been widely utilised for chromosome identification experiments [36]. GISH is a labour-intensive and time-consuming technology relying on fluorescent genome probes that bind to target sequence patterns specific to the corresponding species. The availability of an easy and cost-efficient technology suitable for fast karyotype analysis of different tissue types would be very beneficial for researchers coping with the characterisation of hybrid plants. Since PCR-based marker screening has a higher throughput and is much faster than cytogenetic techniques, DNA marker analysis is the preferred approach for the large-scale karyotyping of hybrid plants. Possible chromosome dosage (e.g., monosomic conditions) and structural rearrangements such as translocation in candidate plants can still subjected to a more in-depth analysis by in situ hybridisation techniques.

An often-neglected advantage of molecular markers over the cytogenetic monitoring of chromosome composition (in interspecific hybrids) is related to the phenomenon of genetic mosaicity. Cytogenetic analysis is performed on individual cells and can identify multiple cells with different chromosome numbers and composition ( [37, 38], also Fig. 6A and C: panel H) within the same tissue sample, which hampers the precise identification of the corresponding individual plant. The DNA template, however, is purified from tissues composed of thousands of cells, therefore the rare mosaic variants are masked or underrepresented in the final DNA (Fig. 6A and C: plant No. 6 vs. Fig. 5: lane 6). Also, the exponential nature of PCR further diminishes the detection of these variants [6].

Whereas the objective of the described MPCR approach was here to characterise the chromosome composition in wheat × barley hybrids the same strategy of primer design will be useful and applicable to many other situations in which interspecific crossing is relevant such as chromosome stability in newly synthetised wheat [39–41] or hybrids in the Brassicas [42].

## Conclusion

This study describes for the first time a multiplex PCR (MPCR) assay optimised to identify individual chromosomes and monitor their composition in wheat × barley hybrids. Large primer sets were designed based on wheat and barley reference genomes and confirmed for specificity *in silico* by alignments with genome sequences of 18 cultivars. Experimental verification was performed with 19 wheat and barley cultivars, as well as 11 *Triticum*, *Aegilops*, and *Hordeum* species. The developed MPCR assay was applied to analyse 16 wheat × barley F1 hybrids and the results were confirmed by the standard GISH technique. The strategy of MPCR primer design can be extended to many plant species with well-characterised and sequenced genomes.

### Electronic supplementary material

Below is the link to the electronic supplementary material.

**Supplementary Material 1:** Genotypes used for primer design, in silico analysis, and MPCR validation

**Supplementary Material 2:** Primers obtained by the refined protocol

**Supplementary Material 3:** The final list of chromosome-specific MPCR primers in the A, B, D, and H (sub-)genomes

**Supplementary Material 4:** In silico prediction of chromosome-specific MPCR products in 16 wheat and two barley genomes

**Supplementary Material 5:** Sequencing results of 28 chromosome-specific PCR products with specific forward and reverse primers

**Supplementary Material 6:** In silico prediction of chromosome-specific MPCR products in the genomes of *Triticum, Aegilops*, and *Hordeum* species

## Data Availability

No datasets were generated or analysed during the current study.

## References

[1] Riley R, Chapman V (1958). Genetic control of the cytologically diploid behaviour of hexaploid wheat. Nature.

[2] Sears ER (1976). Genetic control of chromosome pairing in wheat. Annu Rev Genet.

[3] Tonosaki K, Osabe K, Kawanabe T, Fujimoto R. The importance of reproductive barriers and the effect of allopolyploidization on crop breeding. Breed Sci. 2016;15114. 10.1270/jsbbs.15114 PMC4902455 27436943

[4] McGoverin CM, Snyders F, Muller N, Botes W, Fox G, Manley M (2011). A review of triticale uses and the effect of growth environment on grain quality. J Sci Food Agric.

[5] Molnár-Láng M, Linc G, Szakács É (2014). Wheat–barley hybridization: the last 40 years. Euphytica.

[6] Polgári D, Mihók E, Sági L (2019). Composition and random elimination of paternal chromosomes in a large population of wheat × barley (*Triticum aestivum* L. × *Hordeum vulgare* L.) hybrids. Plant Cell Rep.

[7] Le HT, Armstrong KC, Miki B (1989). Detection of rye DNA in wheat-rye hybrids and wheat translocation stocks using total genomic DNA as a probe. Plant Mol Biology Report.

[8] Schwarzacher T, Leitch AR, Bennett MD, Heslop-Harrison JS (1989). *Situ* localization of parental genomes in a wide hybrid. Ann Botany.

[9] Cseh A, Kruppa K, Molnár I, Rakszegi M, Doležel J, Molnár-Láng M (2011). Characterization of a new 4BS. 7HL wheat–barley translocation line using GISH, FISH, and SSR markers and its effect on the β-glucan content of wheat. Genome.

[10] Szakács É, Kruppa K, Molnár-Láng M (2013). Analysis of chromosomal polymorphism in barley (Hordeum vulgare L. ssp. vulgare) and between H. vulgare and H. chilense using three-color fluorescence in situ hybridization (FISH). J Appl Genet.

[11] Blake TK, Kadyrzhanova D, Shepherd KW, Islam AKMR, Langridge PL, McDonald CL (1996). STS-PCR markers appropriate for wheat-barley introgression. Theor Appl Genet.

[12] Murai K, Taketa S, AKM RI, Shepherd KW (2000). Barley allele-specific amplicons useful for identifying wheat-barley recombinant chromosomes. Genes Genet Syst.

[13] Shan X, Blake TK, Talbert LE (1999). Conversion of AFLP markers to sequence-specific PCR markers in barley and wheat. Theor Appl Genet.

[14] Hagras AAA, Kishii M, Sato K, Tanaka H, Tsujimoto H (2005). Extended application of barley EST markers for the analysis of alien chromosomes added to wheat genetic background. Breed Sci.

[15] Nasuda S, Kikkawa Y, Ashida T, AKM RI, Sato K, Endo TR (2005). Chromosomal assignment and deletion mapping of barley EST markers. Genes Genet Syst.

[16] Ashida T, Nasuda S, Sato K, Endo TR (2007). Dissection of barley chromosome 5H in common wheat. Genes Genet Syst.

[17] Sakai K, Nasuda S, Sato K, Endo TR (2009). Dissection of barley chromosome 3H in common wheat and a comparison of 3H physical and genetic maps. Genes Genet Syst.

[18] Sakata M, Nasuda S, Endo TR (2010). Dissection of barley chromosome 4H in common wheat by the gametocidal system and cytological mapping of chromosome 4H with EST markers. Genes Genet Syst.

[19] Joshi GP, Nasuda S, Endo TR (2011). Dissection and cytological mapping of barley chromosome 2H in the genetic background of common wheat. Genes Genet Syst.

[20] Ishihara A, Mizuno N, Islam RA, Doležel J, Endo TR, Nasuda S (2014). Dissection of barley chromosomes 1H and 6H by the gametocidal system. Genes Genet Syst.

[21] Polgári D, Cseh A, Szakács E, Jäger K, Molnár-Láng M, Sági L (2014). High-frequency generation and characterization of intergeneric hybrids and haploids from new wheat–barley crosses. Plant Cell Rep.

[22] Marçais G, Kingsford C (2011). A fast, lock-free approach for efficient parallel counting of occurrences of k-mers. Bioinformatics.

[23] Prüfer K, Stenzel U, Dannemann M, Green RE, Lachmann M, Kelso J (2008). PatMaN: rapid alignment of short sequences to large databases. Bioinformatics.

[24] Walkowiak S, Gao L, Monat C, Haberer G, Kassa MT, Brinton J (2020). Multiple wheat genomes reveal global variation in modern breeding. Nature.

[25] Fukui K, Kamisugi Y, Sakai F (1994). Physical mapping of 5S rDNA loci by direct-cloned biotinylated probes in barley chromosomes. Genome.

[26] Makai D, Mihók E, Polgári D, Cseh A, Lenykó-Thegze A, Sepsi A. Sági L Rapid in-solution preparation of somatic and meiotic plant cell nuclei for high-quality 3D immunoFISH and immunoFISH-GISH. Plant Methods. 2023;19;80. 10.1186/s13007-023-01061-7 PMC10408160 37553677

[27] Bendich AJ, McCarthy BJ (1970). DNA comparisons among barley, oats, rye, and wheat. Genetics.

[28] Flavell RB, Rimpau J, Smith DB (1977). Repeated sequence DNA relationships in four cereal genomes. Chromosoma.

[29] Rimpau J, Smith D, Flavell R (1978). Sequence organisation analysis of the wheat and rye genomes by interspecies DNA/DNA hybridisation. J Mol Biol.

[30] Rimpau J, Smith DB, Flavell RB (1980). Sequence organisation in barley and oats chromosomes revealed by interspecies DNA/DNA hybridisation. Heredity.

[31] Bilgic H, Cho S, Garvin DF, Muehlbauer GJ (2007). Mapping barley genes to chromosome arms by transcript profiling of wheat–barley ditelosomic chromosome addition lines. Genome.

[32] Zohary D, Feldman M. Hybridization between amphidiploids and the evolution of polyploids in the wheat (*Aegilops*-*Triticum*) group. Evolution. 1962;44–61.

[33] Luo MC, Gu YQ, Puiu D, Wang H, Twardziok SO, Deal KR (2017). Genome sequence of the progenitor of the wheat D genome *Aegilops tauschii*. Nature.

[34] Marcussen T, Sandve SR, Heier L, Spannagl M, Pfeifer M et al. International Wheat Genome Sequencing Consortium,. Ancient hybridizations among the ancestral genomes of bread wheat. Science. 2014;345:1250092. 10.1126/science.1250092 25035499

[35] Huynh S, Marcussen T, Felber F, Parisod C (2019). Hybridization preceded radiation in diploid wheats. Mol Phylogenet Evol.

[36] Schwarzacher T (2016). Preparation and fluorescent analysis of plant metaphase chromosomes. Methods Mol Biol.

[37] Koba T, Handa T, Shimada T (1991). Efficient production of wheat-barley hybrids and preferential elimination of barley chromosomes. Theor Appl Genet.

[38] Taketa S, Kato J, Takeda K (1995). High crossability of wild barley (*Hordeum spontaneum* C. Koch) with bread wheat and the differential elimination of barley chromosomes in the hybrids. Theor Appl Genet.

[39] Zhang H, Bian Y, Gou XW, Zhu B, Xu CM, Qi B (2013). Persistent whole-chromosome aneuploidy is generally associated with nascent allohexaploid wheat. Proc Natl Acad Sci USA.

[40] Li A, Liu D, Yang W, Kishii M, Mao L (2018). Synthetic hexaploid wheat: yesterday, today, and tomorrow. Engineering.

[41] Zhao L, Xie D, Fan C, Zhang S, Huang L, Ning S (2021). Chromosome stability of synthetic-natural wheat hybrids. Front Plant Sci.

[42] Koh JCO, Barbulescu DM, Norton S, Redden S, Salisbury PA, Kaur S, Cogan N, Slater AT (2017). A multiplex PCR for rapid identification of *Brassica* species in the triangle of U. Plant Methods.

