# Correction: Rapid and cost-effective molecular karyotyping in wheat, barley, and their crossprogeny by chromosome-specific multiplex PCR

**DOI:** 10.1186/s13007-024-01179-2

**Published:** 2024-04-20

**Authors:** Mohammad Ali, Dávid Polgári, Adél Sepsi, Levente Kontra, Ágnes Dalmadi, Zoltán Havelda, László Sági, András Kis

**Affiliations:** 1 Institute of Genetics and Biotechnology, Hungarian University of Agriculture and Life Sciences,, 2100 Gödöllő, Hungary; 2 Doctoral School of Plant Sciences, Hungarian University of Agriculture and Life Sciences,, 2100 Gödöllő, Hungary; 3 Centre for Agricultural Research, Hungarian Research Network,, 2462 Martonvásár, Hungary; 4 Agribiotechnology and Precision Breeding for Food Security National Laboratory, Plant Biotechnology Section, Hungarian University of Agriculture and Life Sciences,, 2100 Gödöllő, Hungary; 5 Agribiotechnology and Precision Breeding for Food Security National Laboratory, Plant Biotechnology Section, Centre for Agricultural Research, , 2462 Martonvásár, Hungary; 6 Present Address: Institute of Experimental Medicine, Bioinformatics Core Facility, Hungarian Research Network,, 1083 Budapest, Hungary

**Correction: Plant Methods (2024) 20:37**

10.1186/s13007-024-01162-x

The supplementary material contains unedited figures instead of six tables (Tables S1–S6) . The original article has been corrected.

### Electronic supplementary material

Supplementary Material 1

Supplementary Material 2

Supplementary Material 3

Supplementary Material 4

Supplementary Material 5

Supplementary Material 6

